# Supplementary figures and images for: Genome-wide discovery for diabetes-dependent triglycerides-associated loci
Source: PLoS One. 2022 Oct 21;17(10):e0275934. doi: 10.1371/journal.pone.0275934 (PMC9586367; doi:10.1371/journal.pone.0275934)

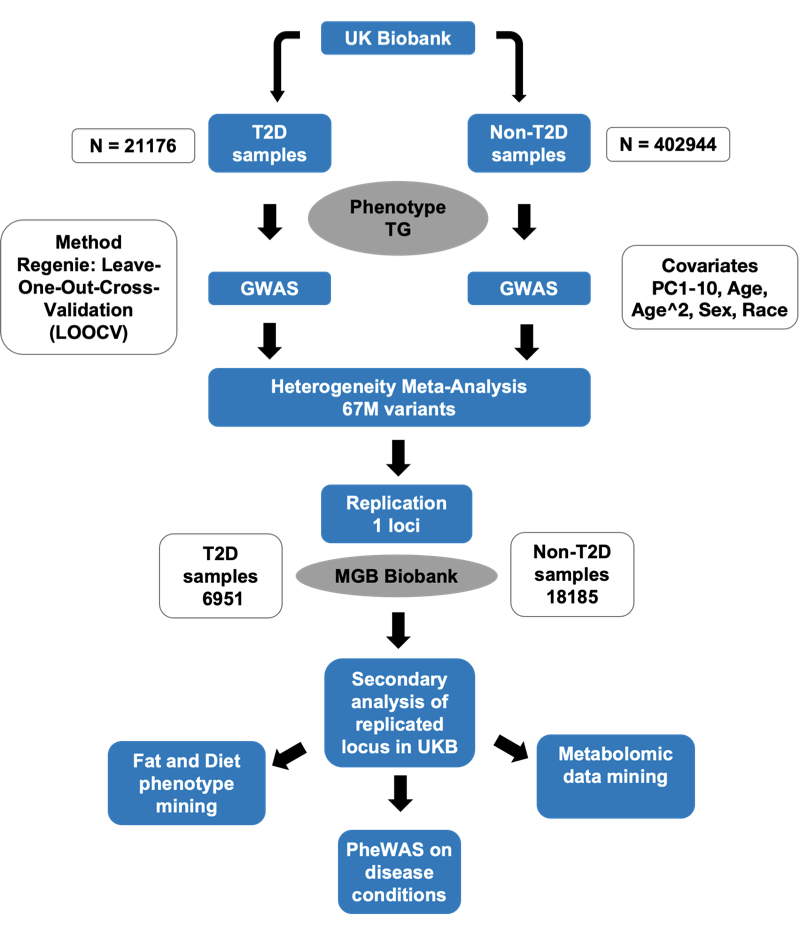

Supplement: S1 Fig — We carried out stratified GWAS on UKB discovery cohort based on T2 status, using Regenie LOOCV models adjusting for age, age2, sex, race, and PC1-10. We implemented heterogeneity analysis to identify loci that was differentially associated between the two strata. Out of the 67M variants analyzed, only one locus achieved genome-wide significance. We replicated the significant locus using MGBB, an independent cohort, and further analyzed the lead variant using various secondary analysis in the discovery cohort. GWAS–Genome wide association; LOOCV—leave-one-out-cross-validation; MGBB–Mass General Brigham Biobank; T2D –Type 2 Diabetes; UKB–UK Biobank. (TIF) [file pone.0275934.s001.tif]

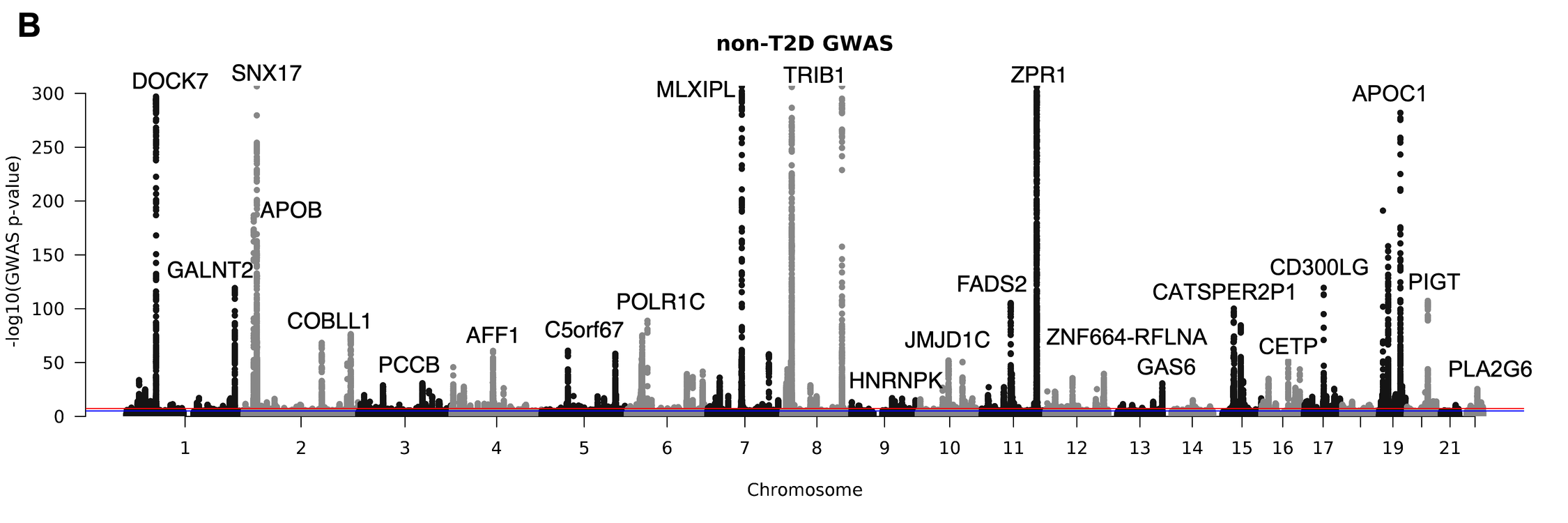

Supplement: S2 Fig — A) MH plots for T2D GWAS B) MH plots for non-T2D GWAS. Genes near to the most significant lead variant in each loci are documented, full list of lead SNPs are tabulated in S1 Table. Red line: Genome significance (p-value = 5x10^-8^), Blue line: Suggestive significance (p-value = 1x10^-5^). GWAS–Genome wide association studies; MH–Manhattan; T2D –Type 2 Diabetes. (ZIP) [file pone.0275934.s002.zip › S2B_Fig.tif]

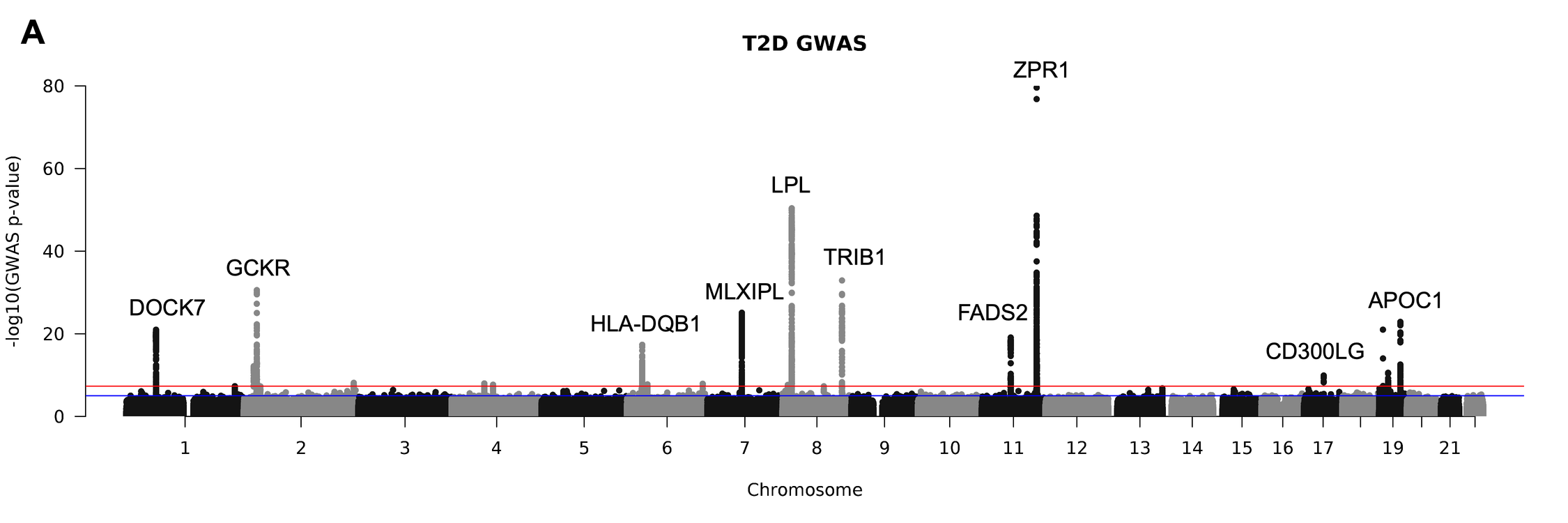

Supplement: S2 Fig — A) MH plots for T2D GWAS B) MH plots for non-T2D GWAS. Genes near to the most significant lead variant in each loci are documented, full list of lead SNPs are tabulated in S1 Table. Red line: Genome significance (p-value = 5x10^-8^), Blue line: Suggestive significance (p-value = 1x10^-5^). GWAS–Genome wide association studies; MH–Manhattan; T2D –Type 2 Diabetes. (ZIP) [file pone.0275934.s002.zip › S2A_Fig.tif]

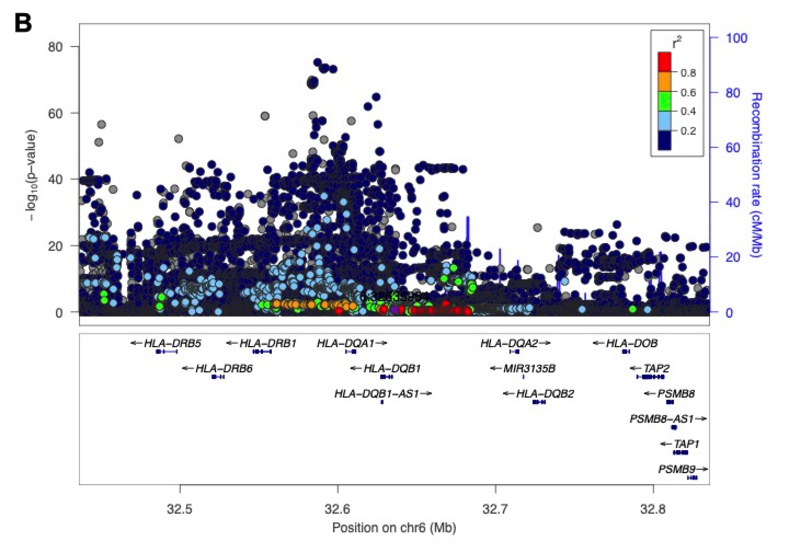

Supplement: S3 Fig — A) Locus zoom plot for HLA-DQB1/DQA2 loci in T2D strata. B) Locus zoom plot for HLA-DQB1/DQA2 loci in non-T2D strata. X-axis defines the genomic position where variants +/-500 kb on either side of rs9274619—chr6:32635954:G:A (grc37) is mapped on the genome. The variants are colored based on the r2 with the lead variant and the genes are mapped based on their genomic position. Y-axis is the -log10(p-values) from the respective strata and the scale of y-axis is different between the two plots. HLA–Human Leukocyte Antigen; T2D –Type 2 Diabetes. (ZIP) [file pone.0275934.s003.zip › S3B_Fig.tif]

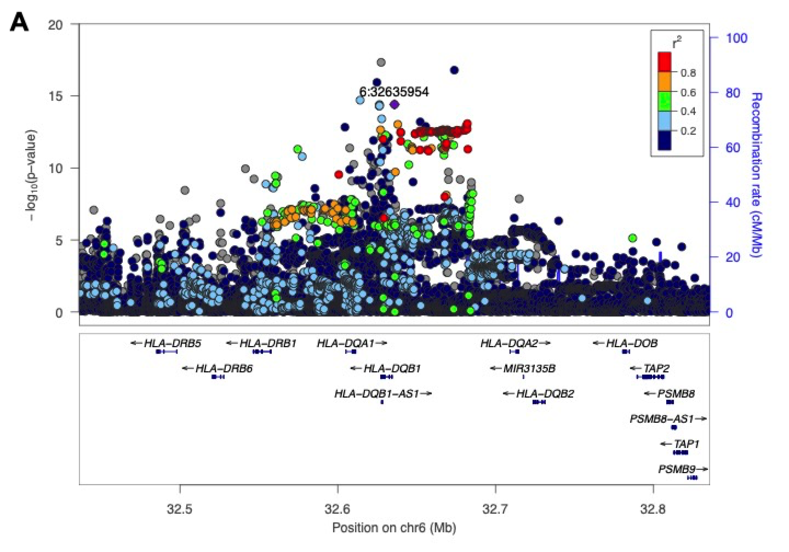

Supplement: S3 Fig — A) Locus zoom plot for HLA-DQB1/DQA2 loci in T2D strata. B) Locus zoom plot for HLA-DQB1/DQA2 loci in non-T2D strata. X-axis defines the genomic position where variants +/-500 kb on either side of rs9274619—chr6:32635954:G:A (grc37) is mapped on the genome. The variants are colored based on the r2 with the lead variant and the genes are mapped based on their genomic position. Y-axis is the -log10(p-values) from the respective strata and the scale of y-axis is different between the two plots. HLA–Human Leukocyte Antigen; T2D –Type 2 Diabetes. (ZIP) [file pone.0275934.s003.zip › S3A_Fig.tif]

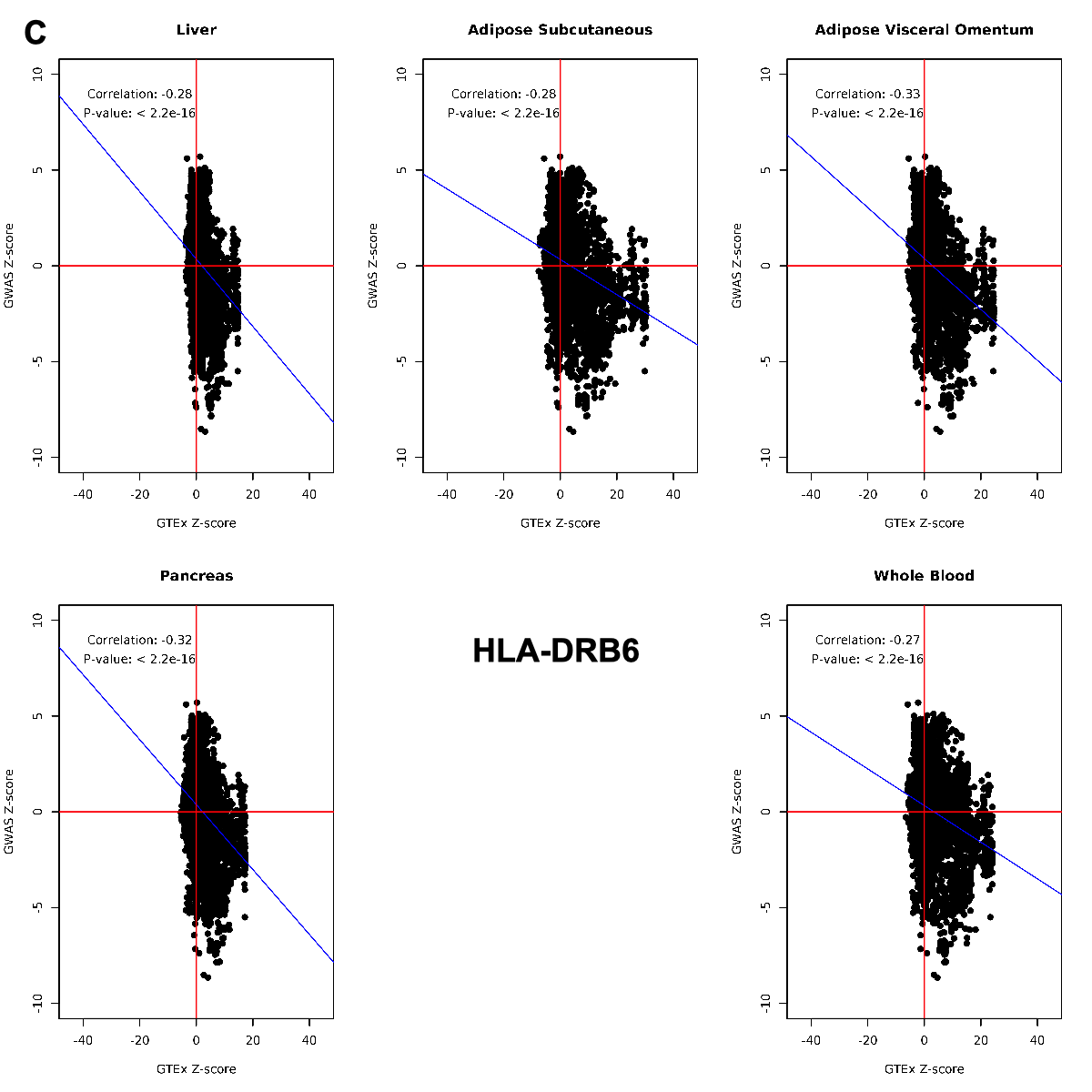

Supplement: S4 Fig — Z-scores were calculated from T2D GWAS and GTEx (version 8) summary statistics for all the eQTLs for the three genes. Pearson correlation coefficient was calculated, and scatter plots were generated for eQTL data from five different tissues. Most of the TG lowering variants increases the expression of HLA-DQA2/HLA-DRB6, whereas decreases the expression of HLA-DQB1. (ZIP) [file pone.0275934.s004.zip › S4C_Fig.tif]

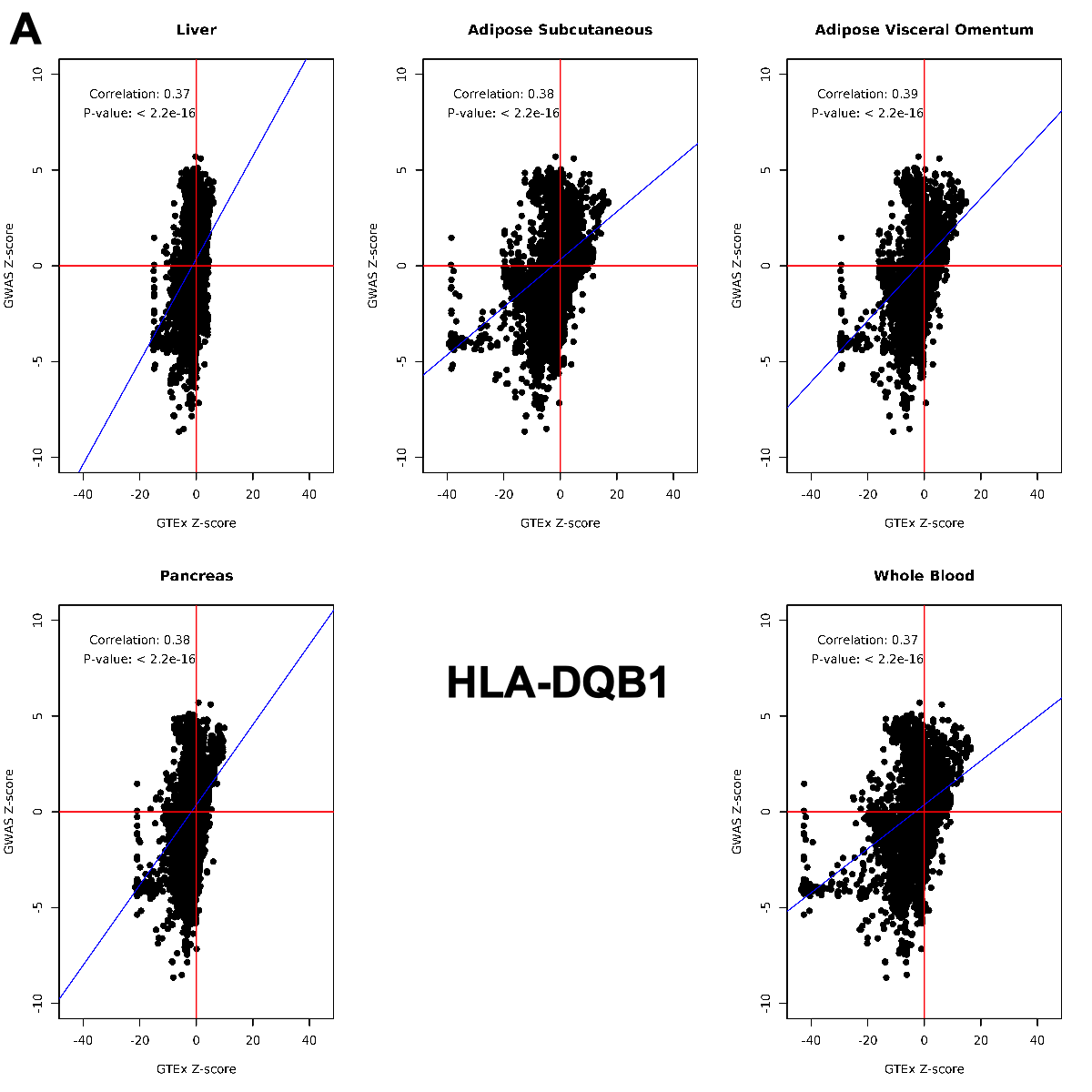

Supplement: S4 Fig — Z-scores were calculated from T2D GWAS and GTEx (version 8) summary statistics for all the eQTLs for the three genes. Pearson correlation coefficient was calculated, and scatter plots were generated for eQTL data from five different tissues. Most of the TG lowering variants increases the expression of HLA-DQA2/HLA-DRB6, whereas decreases the expression of HLA-DQB1. (ZIP) [file pone.0275934.s004.zip › S4A_Fig.tif]

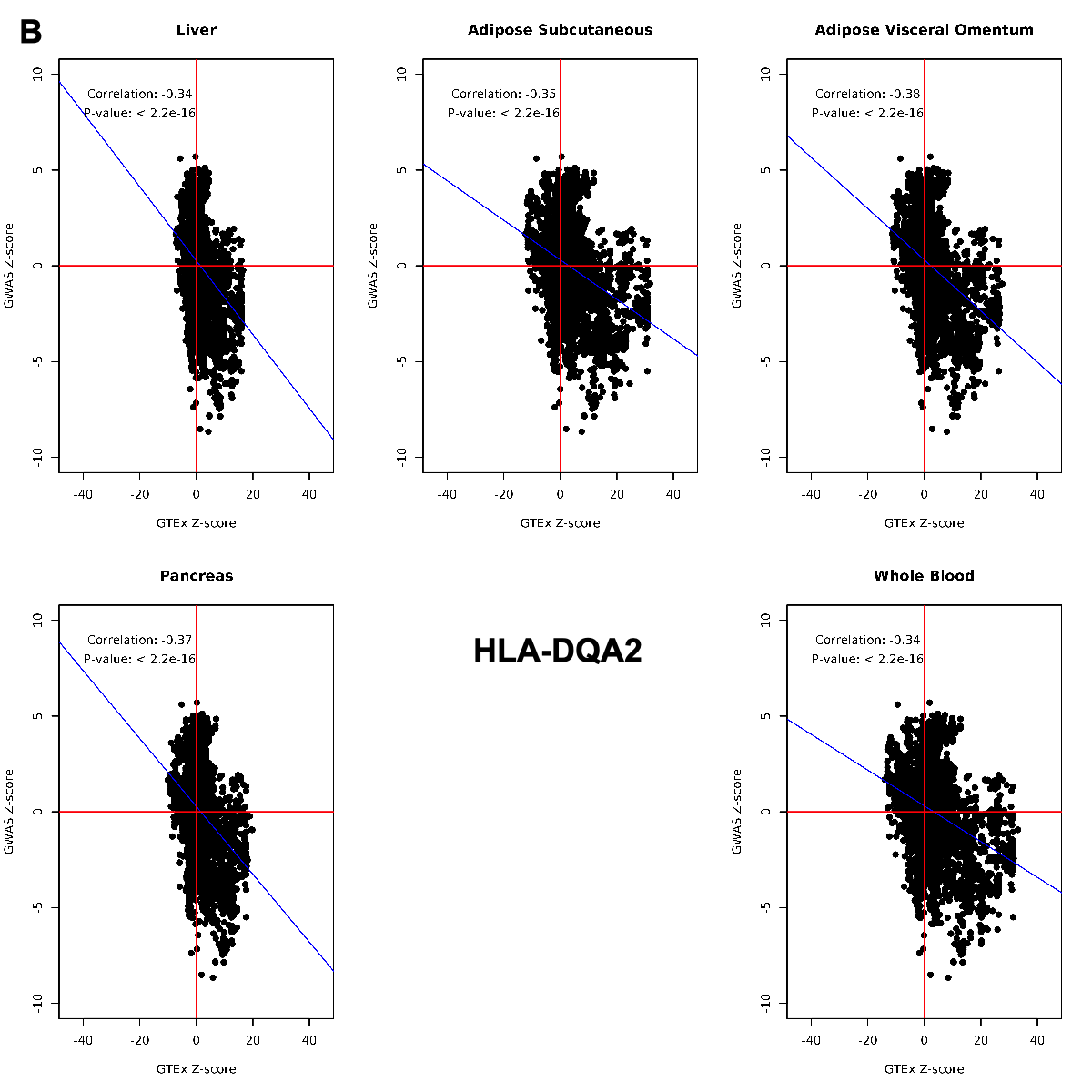

Supplement: S4 Fig — Z-scores were calculated from T2D GWAS and GTEx (version 8) summary statistics for all the eQTLs for the three genes. Pearson correlation coefficient was calculated, and scatter plots were generated for eQTL data from five different tissues. Most of the TG lowering variants increases the expression of HLA-DQA2/HLA-DRB6, whereas decreases the expression of HLA-DQB1. (ZIP) [file pone.0275934.s004.zip › S4B_Fig.tif]

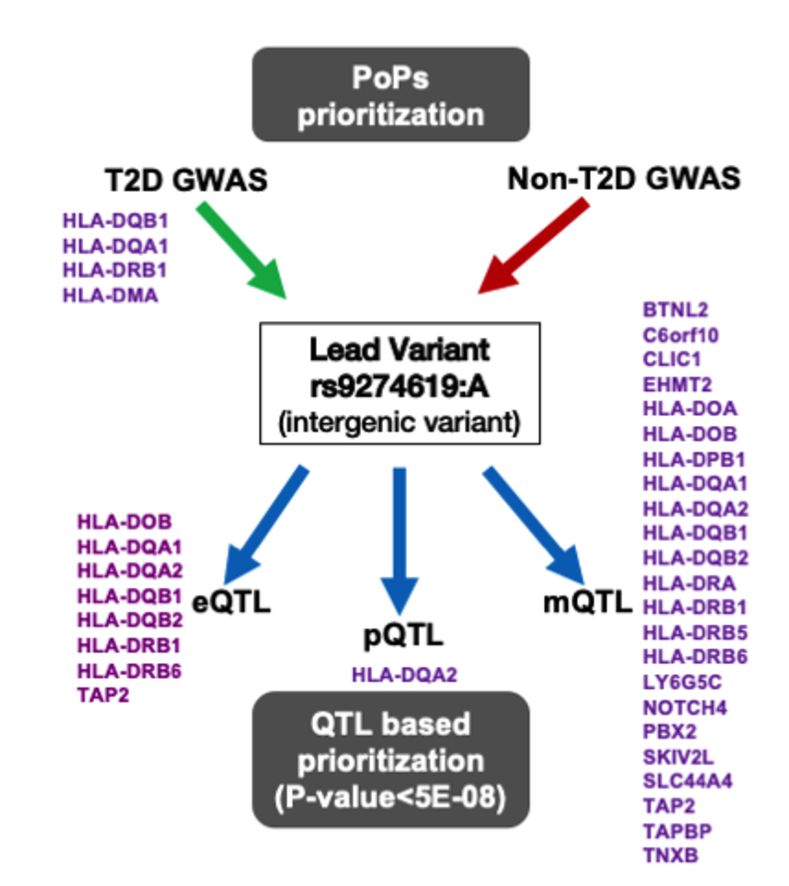

Supplement: S5 Fig — PoPS method was used to prioritize genes using GWAS summary statistics from both T2D and non-T2D stratum. Multiple HLA genes were prioritized, where HLA-DQB1 topped the list. eQTL, pQTL and mQTL curation of rs9274619:A from various public repositories mapped the lead variant to multiple HLA-genes, where HLA-DQA2 was identified by all three QTL searches. (TIF) [file pone.0275934.s005.tif]

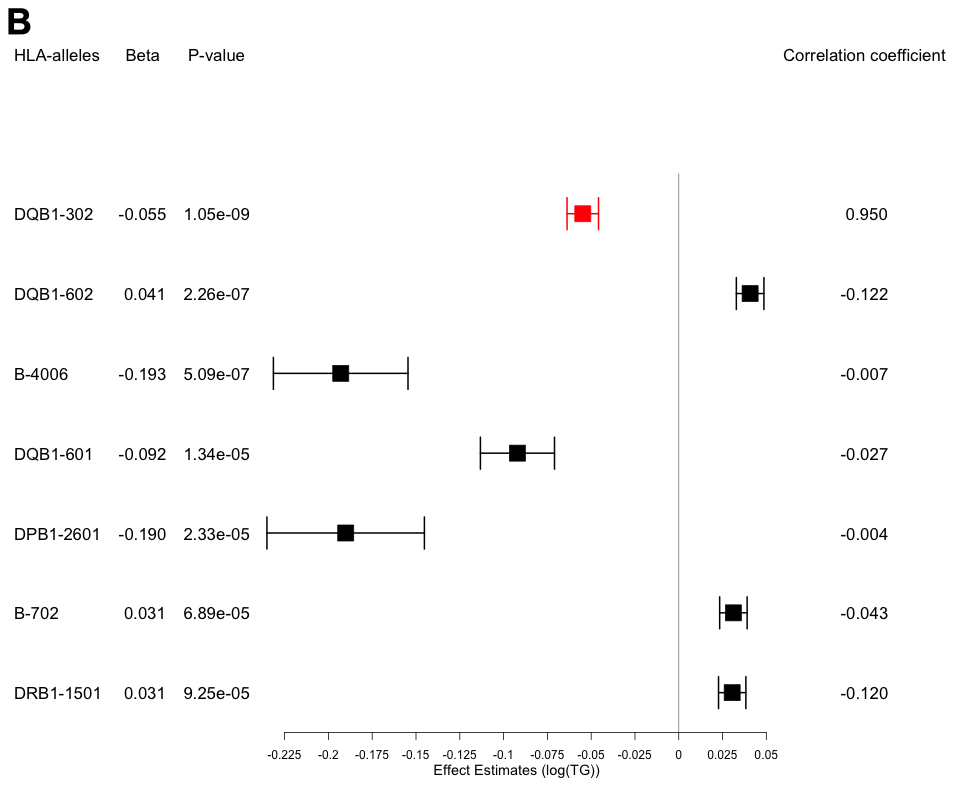

Supplement: S6 Fig — A) Correlation between rs9274619:A and HLA alleles in DQB1 and DQA1 class. DQB1-302 is the most strongly correlated allele. B) Forest plot showing the different alleles that passed the Bonferroni correction on interacting with T2D with log(TG) as outcome, the model was adjusted age, age2, sex, race, PC1-10 and rs9274619:A. DQB1-302 allele is the only allele with significant interaction with T2D and highly correlated to rs9274619:A (mapped in red). HLA–Human Leukocyte Antigen; T2D –Type 2 Diabetes; TG–Triglycerides; VOI–Variant of interest. (ZIP) [file pone.0275934.s006.zip › S6B_Fig.tif]

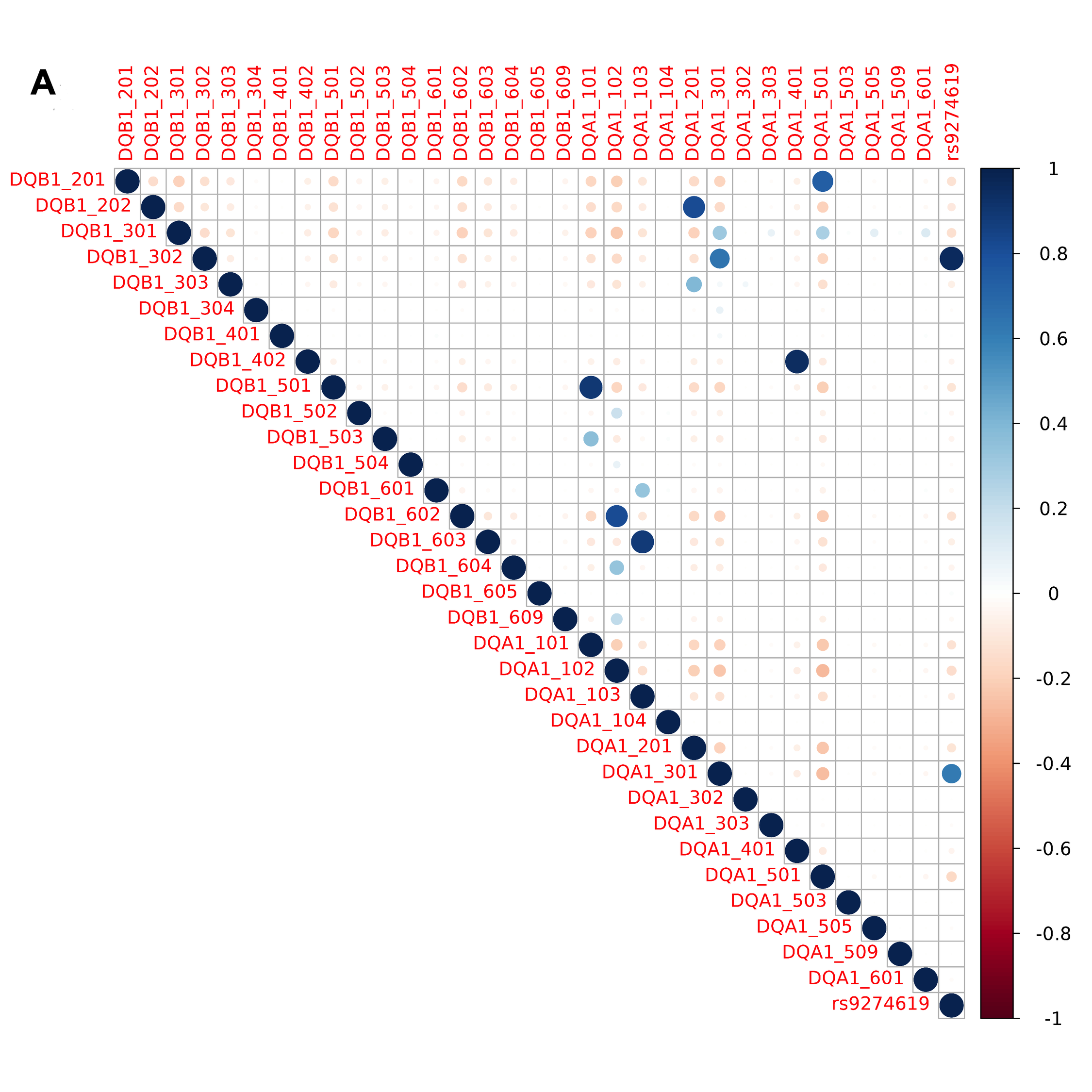

Supplement: S6 Fig — A) Correlation between rs9274619:A and HLA alleles in DQB1 and DQA1 class. DQB1-302 is the most strongly correlated allele. B) Forest plot showing the different alleles that passed the Bonferroni correction on interacting with T2D with log(TG) as outcome, the model was adjusted age, age2, sex, race, PC1-10 and rs9274619:A. DQB1-302 allele is the only allele with significant interaction with T2D and highly correlated to rs9274619:A (mapped in red). HLA–Human Leukocyte Antigen; T2D –Type 2 Diabetes; TG–Triglycerides; VOI–Variant of interest. (ZIP) [file pone.0275934.s006.zip › S6A_Fig.tif]
