# Supplementary material for: Genome-wide discovery for diabetes-dependent triglycerides-associated loci
Source: PLoS One. 2022 Oct 21;17(10):e0275934. doi: 10.1371/journal.pone.0275934 (PMC9586367; doi:10.1371/journal.pone.0275934)
Supplement: Attachment — Submitted filename: Response_to_Reviewers_AfterRevision.docx [file pone.0275934.s024.docx]

**Editors Comment:**

Please review your reference list to ensure that it is complete and correct. If you have cited papers that have been retracted, please include the rationale for doing so in the manuscript text, or remove these references and replace them with relevant current references. Any changes to the reference list should be mentioned in the rebuttal letter that accompanies your revised manuscript. If you need to cite a retracted article, indicate the article’s retracted status in the References list and also include a citation and full reference for the retraction notice.

Authors response:

Thank you for the comment. We have corrected one reference and removed one reference.

Reference no 14:

Young KG, McDonald TJ, Shields BM. Glycated haemoglobin measurements from UK Biobank are different to those in linked primary care records: implications for combining biochemistry data from research studies and routine clinical care. Int J Epidemiol. 2022 Jun 13;51(3):1022–4.

Reference no 27:

Removed

**Reviewers' comments:**

Reviewer #1: This study highlights the necessity of understanding the genetics of T2D and underlying pathways.
T2D is heterogeneous complex genetic trait, in order to proceed for personalized medical care for each patient, tailored medical care based on genetics, genetic risk scores offers valuable tool for patient T2D characterization and drug _repurposing.

Authors response:

Thank you for the positive comment.

Reviewer #3: The authors have answered my queries. It would strengthen the observed association to check if TG levels are related to the rs9274619 genotypes A/A, A/G and G/G. As rs9274619 is located between the HLA-DQB1 and HLA-DQA2 loci, the authors may want to refer this potentially quantitative trait loci as linked to HLA-DQB1/HLA-DQA2 rather than only HLA-DQB1.
LD to either gene may be about the same.

The authors should consider to add this recent investigation of small vessel vasculitis, an immune related disease also related to rs9274619: Dahlqvist et al. Identification and functional characterization of a novel susceptibility locus for small vessel vasculitis with MPO-ANCA. Rheumatology (Oxford). 2022 Aug 3;61(8):3461-3470.

Finally, HLA genotypes seemed to influence levels of TG in children at increased HLA genetic risk and positive for islet autoantibodies but prior to clinical diagnosis. TG levels were lower in the not-yet diabetes affected subjects and may indeed reflect the rs9274619 variant.

Authors response:

Thank you for the suggestion, we have added the below sentences to address the comments. Throughout the manuscript we have made changes to sentences referring the lead variants proximity to *HLA-DQB1/DQA2*. Additionally, we agree that our results showing the main effect of rs9274619 on TGs is consistent with the prior literature and here we show its dependency on diabetes status.

Line number 114: The mean raw TG measurements were significantly different between the reference and alternative genotypes in T2D samples (rs9274619(G/A or A/G) : mean_diff_=5.45 mg/dl; p-value=1.26x10^-02^; rs9274619(A/A) : mean_diff_=25.6 mg/dl; p-value=3.78x10^-03^).

Line number 165: Since rs9274619:A located between both *HLA-DQB1* and *HLA-DQA2*, the variant could be a potential quantitative trait loci to both the genes.

Line number 193: Interestingly, a recent investigation on Anti-neutrophil cytoplasmic antibody (ANCA)-associated vasculitides (AAV) by Dahlqvist et al have identified rs9274619 as a lead variant for myeloperoxidase (MPO)-ANCA association (11), showing its importance in immune-related vascular diseases.

**Figures:**

While revising your submission, please upload your figure files to the Preflight Analysis and Conversion Engine (PACE) digital diagnostic tool, [https://pacev2.apexcovantage.com/](https://secure-web.cisco.com/1Zn1LuHMlnL59ddsLGiLMoyZHvYV4FVrPdWwkoi-N1HrDlmnjC5RxVIIzXVcHmgD-VzVBSbFqzE1dwHMn8gCVq63O3koj_5tM0VeaJqO2Et1Wttpuw3rQtrULFpyCdZEMxMa732wy60lZ-lflZJn8vzs8i8z7EdylYB-R88aq9tw-JVRj-sbgSLKoEMw43de82i8TfEn9SVej69Kc6Y35-Iy1ZSJgrHr4QheQAzFQeEe_AIUql1k9A0Gl33fFkvTdafEdRy-cfjfhZEFQrSJ4NX8Prde7ANzGR6LqvUya2VBwkaXXvWpI1HKT94mb2luZ/https%3A%2F%2Fpacev2.apexcovantage.com%2F). PACE helps ensure that figures meet PLOS requirements. To use PACE, you must first register as a user. Registration is free. Then, login and navigate to the UPLOAD tab, where you will find detailed instructions on how to use the tool. If you encounter any issues or have any questions when using PACE, please email PLOS at [figures@plos.org](mailto:figures@plos.org). Please note that Supporting Information files do not need this step.

Authors response:

Thank you for the information. We have uploaded the figures through this tool.
